# The Love of God to Man

**Published:** 1889-08-03

**Authors:** 


### Words of Consolation.

### THE LOVE OF GOD TO MAN.

(*For Reading to the Sick.*)

Love, joy, peace. Who would not buy such happiness at any price? Silver and gold are as nothing to the man who can possess himself of such treasures. And yet they are not sought for as one would have imagined, though offered to us "without money and without price." "The fruit of the Spirit is love, joy, peace;" why do we not stretch forth the hand and pluck them? Alas, for the blindness of man's heart!

Love is one of the attributes of the Father, Who created us after His own image, Who redeemed us by the precious blood of His dear Son, and Who showers countless blessings on us every day of our lives, and gives His angels charge over us to keep us in all our ways. What comfort, what bliss to know that in sickness and health, in riches and poverty, in tribulation and joy alike, we have a loving Father whose ears are open to all that ask in His Son's name.

If our trials and sufferings in this life are bitter, we should rejoice, inasmuch as we are more like to Him "Who bore our griefs and carried our sorrows," and Who has given us the welcome invitation "Come unto Me all that travail and are heavy-laden and I will refresh you." Having suffered for man, He is full of sympathy for our woes and in mercy will not send on us more than we can bear, whether it be pains of body or trouble of mind with which He afflicts us. St. Peter says "Beloved, think it not strange concerning the fiery trial which is to try you, as though some strange thing happened unto you. But rejoice, inasmuch as ye are partakers of Christ's sufferings, that when His glory shall be revealed, ye may be glad also with exceeding joy."

God is full of love, giving us more than either we desire or deserve, and extending this loving care to all things of His creation, for a sparrow falleth not to the ground without His knowledge. Man may therefore confidently trust in His merciful goodness, since out of boundless love, which would not allow the work of His hands to be lost, the Eternal Word left the bosom of the Father to obtain man's salvation.

Be assured this merciful God will not send on us more than we can bear, whether it be pain of body or of mind, with which we are afflicted. Fix, then, O suffering soul, thy whole faith and trust on the dear Lord Who bought thee; the parched tongue, the aching head, shall be cooled by the rivers of water which flow from the throne of our God, the trembling limb, the aching heart, shall be strengthened by the fire of His love. Cast all thy burdens on Him Who careth for thee, and He will lead thee safely through the tangled wilderness of this world into "pastures fair by pleasant streams," where He will feed thee with the Bread of Life, and fit thee for the eternal joys of heaven.

"God only knows the love of God;
O, that it now were shed abroad
In this poor stony heart!
For love I sigh, for love I pine;
This only portion, Lord, be mine,
Be mine this better part."

